# The TRPA1 cation channel is upregulated by cigarette smoke in mouse and human macrophages modulating lung inflammation

**DOI:** 10.1038/s41598-025-95662-y

**Published:** 2025-03-27

**Authors:** Anita Steib, Katalin Rozmer, Éva Szőke, József Kun, Nelli Farkas, Diána Feller, Judit Pongrácz, Krisztina Pohóczky, Zsuzsanna Helyes

**Affiliations:** 1 Department of Pharmacology and Pharmacotherapy, Medical School, University of Pécs, Pécs, Hungary; 2 Hungarian Research Network, Chronic Pain Research Group (HUN-REN PTE), Pécs, Hungary; 3 Institute of Pharmaceutical Chemistry, Faculty of Pharmacy, University of Pécs, Pécs, Hungary; 4 National Laboratory for Drug Research and Development, Budapest, Hungary; 5 Hungarian Centre for Genomics and Bioinformatics, Szentágothai Research Centre, University of Pécs, Pécs, Hungary; 6 Institute of Bioanalysis, Medical School, University of Pécs, Pécs, Hungary; 7 Department of Pharmaceutical Biotechnology, Faculty of Pharmacy, University of Pécs, Pécs, Hungary; 8 Department of Pharmacology, Faculty of Pharmacy, University of Pécs, Pécs, Hungary; 9 PharmInVivo Ltd., Pécs, Hungary

**Keywords:** TRPA1, Alveolar macrophage, Cigarette smoke exposure, TGFβ, Airway inflammation, 3D human lung spheroid, Respiration, Respiratory tract diseases, Target identification

## Abstract

Cigarette smoke (CS) is a well-known source of several inflammatory, cytotoxic and genotoxic compounds that cause chronic lung diseases. The transient receptor potential ankyrin 1 (TRPA1), a smoking-responsive, non-selective cation channel, is expressed by both capsaicin-sensitive peptidergic sensory nerves and non-neuronal cells of the lung, but there are few and controversial data on its expression and function on macrophages. Here, we investigated TRPA1 mRNA and protein expression in mouse and human lung tissues and human 3D spheroids, with a particular focus on its expression and potential regulatory effects on pro- and anti-inflammatory macrophage functions in response to CS. TRPA1 was stably expressed in both human and mouse alveolar macrophages, being upregulated after CS exposure and its functional activity was demonstrated in mouse macrophage culture. Moreover, besides CS, the TRPA1 genotype itself affected the expression of M1- (Il-1β, Il-23) and M2-type (Il-10, Tgfβ) macrophage cytokines. Furthermore, CS extract increased TRPA1 mRNA in human lung spheroids showing more prominent expression in macrophage-containing 3D aggregates, while CS extract influenced an elevated TGFβ expression specifically in macrophage-containing spheroids. These results suggest the fine-tuning role of TRPA1 activation in CS-induced airway inflammation, particularly in macrophages, but further studies are needed to draw precise conclusions.

**Supplementary Information:**

The online version contains supplementary material available at 10.1038/s41598-025-95662-y.

## Introduction

The transient receptor potential ankyrin 1 (TRPA1) non-selective cation channel can be activated by a hallmark of irritants and air-borne pollutants such as allyl-isothiocyanate (AITC)^1^, cinnamaldehyde^2^, hydrogen-sulfide^3^, chlorine^4^, aldehydes (formaldehyde, crotonaldehyde)^5,6^ and cigarette smoke (CS) containing polycyclic aromatic hydrocarbons and acrolein^7,8^. The activation of TRPA1 by these compounds has been shown to have an impact on gene expression, secretion of bioactive substances, cell proliferation and survival^9,10^.

TRPA1 is expressed predominantly on sensory neurons and various non-neuronal cells^11^ including dendritic cells, T-lymphocytes, neutrophil granulocytes, and mast cells^12–15^. TRPA1 immunopositivity was also described in macrophages infiltrating human ectopic endometrial tissue^16^, its mRNA and protein expressions were demonstrated in human and mouse colon samples^17^, human oral submucosa^18^, nasal polyps of chronic rhinosinusitis^19^, and primary monocytes^20^, while its mRNA expression was confirmed in murine peritoneal and cutaneous macrophages^21–23^.

TRPA1 is expressed and functionally active on peptidergic sensory nerves innervating the entire respiratory tract^24^ and its activation induces protective reflexes such as apnea, bradycardia, coughing, sneezing and mucus secretion^25–27^. Non-neuronal expression has also been described on lung fibroblasts^28^, lymphocytes^29^, alveolar^30^, bronchial^31^ epithelial and smooth muscle cells^32^. Alveolar macrophages are located on the luminal surface of the alveolar space being the first line of defense against respiratory pathogens and pollutants. CS is a common environmental stimulus that macrophages are exposed to^33^. Cigarette smoke exposure (CSE) induces inflammation and modulates macrophage phenotype and function^34^. Activated macrophages can be divided into two distinct subgroups: pro-inflammatory (killing) M1 and anti-inflammatory (repairing) M2 macrophages. M1 macrophages show increased antigen presentation, inhibiting cell proliferation and damaging tissues, with simultaneous production of nitric oxide, reactive oxygen species and several inflammatory cytokines including interleukins (IL)-1β and -23. M2 macrophages show immunosuppressive behavior with poor antigen presentation, cell proliferation, wound healing and angiogenesis with the secretion of anti-inflammatory cytokines like transforming growth factorβ (TGFβ) and IL-10^35^. Cytokines are not produced exclusively by macrophages, but also by T-lymphocytes, mast cells, stromal cells, epithelial cells, neutrophils^36^.

Although the expression of several TRP channels has been described previously in human and mouse alveolar macrophages^37–39^, TRPA1 has not been detected in such cells^40^. Interestingly, acrolein, a potent agonist of TRPA1 induces IL-8 and tumor necrosis factorα (TNFα) release of primary cultures of human alveolar macrophages obtained by BAL of adult males undergoing diagnostic bronchoscopy for lung cancer^41^.

CS contains α,β-unsaturated aldehydes such as acrolein and crotonaldehyde, which activate TRPA1^42–44^. Gaseous CS or CS extract stimulates Ca^2+^ influx through the activation of TRPA1 channels of primary human airway smooth muscle cells, which shows an important link between TRPA1, smoking and airway hyperresponsiveness^32^. Acrolein induces TRPA1 expression of A549 lung carcinoma cell line at transcriptional level by the activation of several transcriptional factors and mediates acrolein cytotoxicity^44^. In in vitro experiments CS extract induced lung epithelial cell damage on human A549 cells through TRPA1^45^, and TRPA1 has been shown to mediate airway hyperresponsiveness and lung inflammation induced by fine particulate matter in vivo^46^. CS, crotonaldehyde and acrolein induced the contraction of isolated guinea pig bronchi and promoted Ca^2+^ influx in guinea pig jugular ganglia neurons in vitro, which was abolished by the selective TRPA1 antagonist HC-030031 and aldehyde scavenger glutathione. The same prohibiting effect on CSE-induced Ca^2+^ influx in *Trpa1*^−/−^ mice dorsal root ganglion neurons was observed^47^. CSE-induced TRPA1 upregulation of lung epithelial cells in wild-type mice, and airway inflammation was reduced in *Trpa1*^−/−^ mice^48^.

The CSE-induced chronic airway inflammation mouse model was first characterized and the 3-dimensional (3D) human lung tissue models were first set up by our research group. In the present study we aimed to determine the role of TRPA1 in CS-induced airway inflammation in mice and human 3D pulmonary cultures with special emphasis on macrophages.

## Results

### TRPA1 mRNA is expressed on mouse alveolar macrophages

**Fig. 1:**
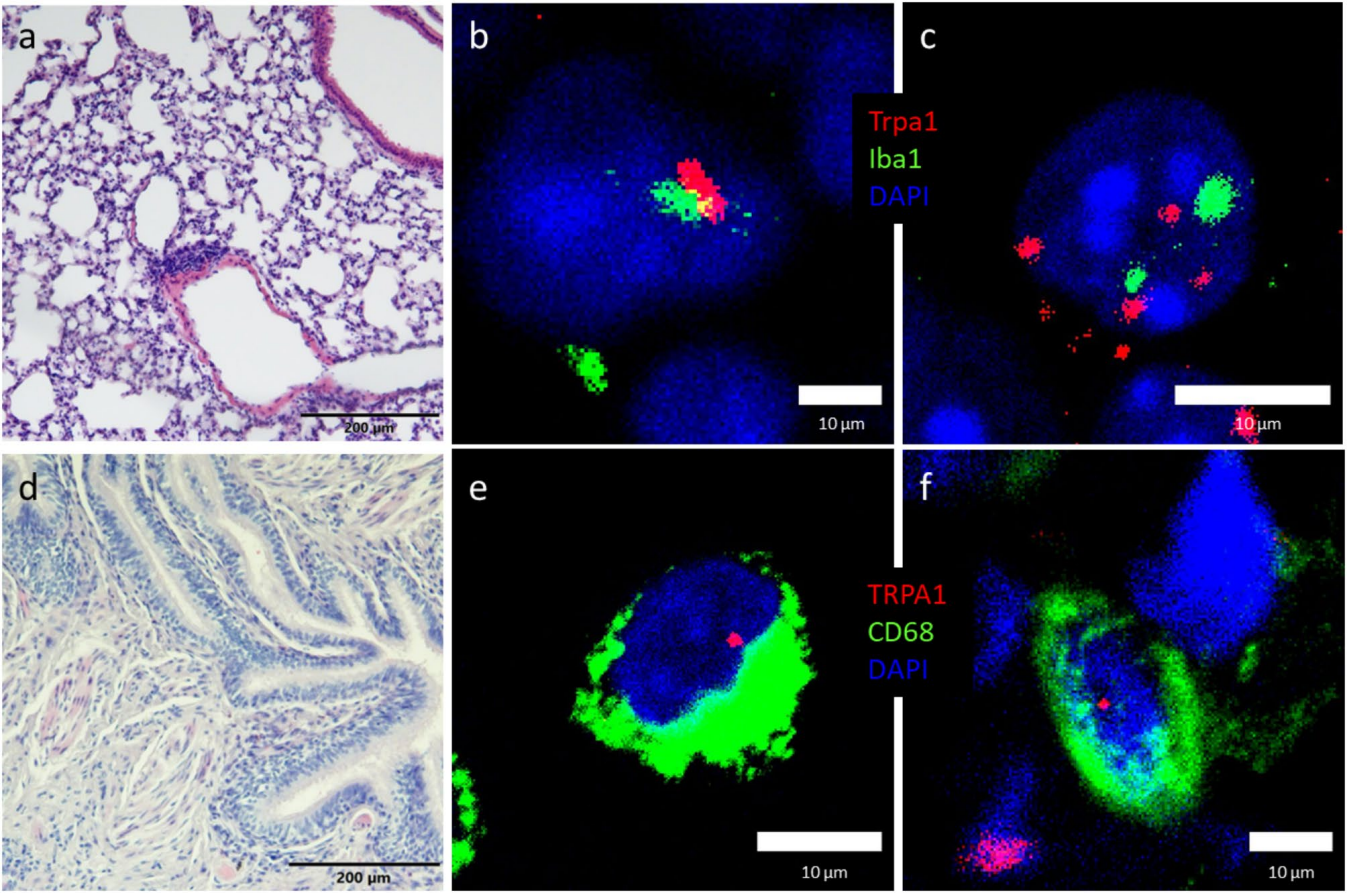
TRPA1 mRNA expression in mouse and human alveolar macrophages. Representative images of hematoxylin–eosin staining of non-smoking healthy mouse (**a**) and human (**d**) lung tissue representing surface epithelium, supporting tissue and network of capillaries (Scale bar: 200 µm), RNAscope images of alveolar macrophages co-expressing *Trpa1* (red signal) and *Iba1* (green signal) in the mouse (**b**, **c**), and *TRPA1* (red signal) and CD68 (green signal) in the human lung (**e**, **f**). Scale bars of (**b**, **c**, **e**, **f**): 10 µm.

To demonstrate the tissue distribution and cell-specific expression of TRPA1 mRNA in the lung, a highly sensitive and specific *RNAscope *in situ hybridization (ISH) has been performed. TRPA1 mRNA was visualized on mouse (Fig. 1b,c) and human (Fig. 1e,f) alveolar macrophages by RNAscope ISH. *Trpa1* was observed in the mouse macrophages in co-localization with *Iba1*, while in the human lung, *TRPA1* was co-expressed with CD68, detected by immunohistochemistry. To control the localization of mouse and human lung tissue sections haematoxylin–eosin staining has been performed (Fig. 1a,d).

Remarkable TRPA1 mRNA expression was detected in the CD45 + cell population of the intact mouse lung related to alveolar macrophages compared to the whole lung, as well as the positive controls trigeminal ganglia and nasal mucosa (Suppl. Figure 1).

### Functional TRPA1 is expressed on mouse macrophages

**Fig. 2:**
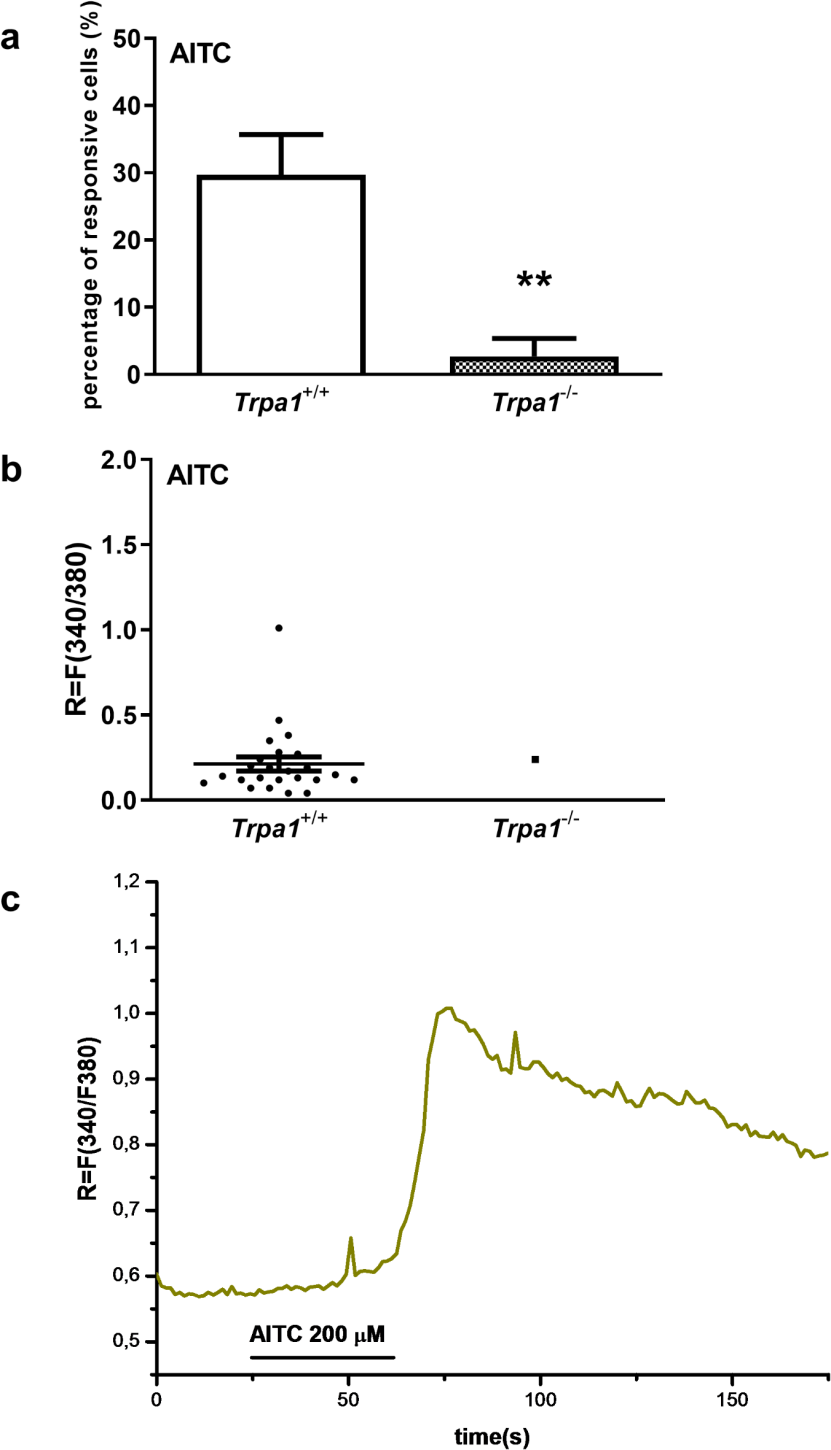
Effect of AITC (200 μM) on cultured mouse macrophages. (**a**) The percentage of responsive cells after AITC administration (200 μM) is presented in % of total number of examined cells, **g > 0.8, (*Trpa1*^+/+^ vs. *Trpa1*^−/−^, effect size analysis, n = 98 cells). (**b**) Change of the fluorescence ratio (R = F340/F380) after AITC administration (*Trpa1*^+/+^ n = 24, *Trpa1*^−/−^ n = 1). Each column represents mean ± SEM. (**c**) Representative original recording showing the response induced by AITC on a wildtype mouse macrophage.

To demonstrate TRPA1 functional activity on mouse peritoneal macrophages Ca^2+^-influx assay has been performed. On the cultured macrophages, the TRPA1 activator AITC (200 μM) evoked a Ca^2+^-influx after 30 s latency providing evidence for the presence of the functional receptor protein in these cells. The percentage of AITC-responsive cells was 29.6 ± 6% (29 out of 98), the R value was 0.21 ± 0.04 (g: 1.66, effect size analysis, Fig. 2a,b). The specificity of the reaction was confirmed using *Trpa1*^−/−^ macrophages from which 2.66% (1 out of 38) responded to AITC treatment (Fig. 2). No Ca^2+^ influx was detected in response to 0.2% (v/v) DMSO as vehicle control.

### CSE-induced macrophage infiltration is TRPA1-mediated

**Fig. 3:**
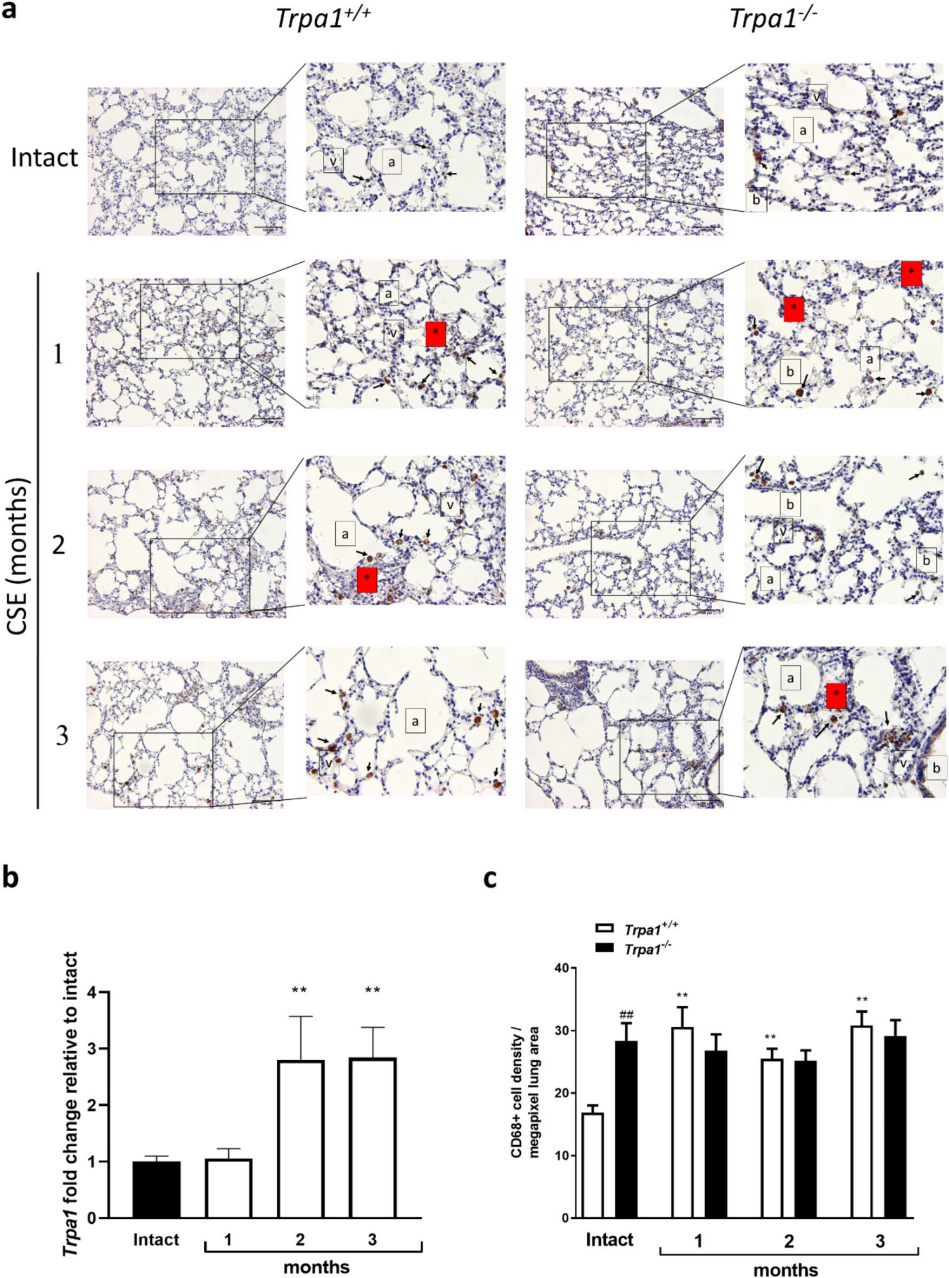
Effects of chronic cigarette smoke exposure on *Trpa1*^+/+^ and *Trpa1*^−/−^ mouse lungs. (**a**) Representative images of macrophage-specific CD68 immunostaining counterstained with hematoxylin of the intact and inflamed mouse lungs after 1, 2 and 3 months of CSE (Magnification: 200x, arrows: CD68 immunpositivity, a: alveolus, b: bronchiolus, v: vessel, red boxes with *: oedema) (**b**) TRPA1 mRNA expression changes of *Trpa1*^+/+^ mouse lungs after 1, 2 and 3 months of CSE. Each column represents mean ± SEM, n = 5–9/group, *g > 0.5, **g > 0.8, vs. *Trpa1*^+/+^ intact, effect size analysis. (**c**) Evaluation of CD68 positive cell density in *Trpa1*^+/+^ and *Trpa1*^−/−^ mouse lungs after CSE. Each column represents mean ± SEM, n = 5 tissue sections/group, 20 images/section; *g > 0.5, **g > 0.8, * vs. respective control, #g > 0.5, ##g > 0.8, # vs. respective treatment, effect size analysis.

To investigate the involvement of TRPA1 in chronic CSE-induced airway inflammation and macrophage infiltration, the lung samples of *Trpa1*^+/+^ and *Trpa1*^−/−^ mice were histopathologically analyzed. After 3 months of smoking peribronchial, perivascular oedema formation and inflammatory cell accumulation with spreading into the interstitial spaces and mild emphysema were observed similarly in both genotypes (Fig. 3a).

TRPA1 mRNA was upregulated with moderate effect size in the mouse lung after 2 months of CSE in the *Trpa1*^+/+^ animals compared to intact animals, and remained elevated at the 3-month timepoint as well (Fig. 3b, suppl. Table 1).

CD68 immunopositivity demonstrating alveolar macrophages was greater in the non-inflamed lung of *Trpa1*^−/−^ mice with large effect size compared to *Trpa1*^+/+^ animals. The CD68 immunopositivity increased with large effect size values in response to 1, 2 and 3 months of CSE in the lung of *Trpa1*^+/+^, but not in *Trpa1*^−/−^ mice (Fig. 3c, Suppl. Table 2).

### CS induces expression changes of M1- and M2-type cytokines in the mouse lung

**Fig. 4:**
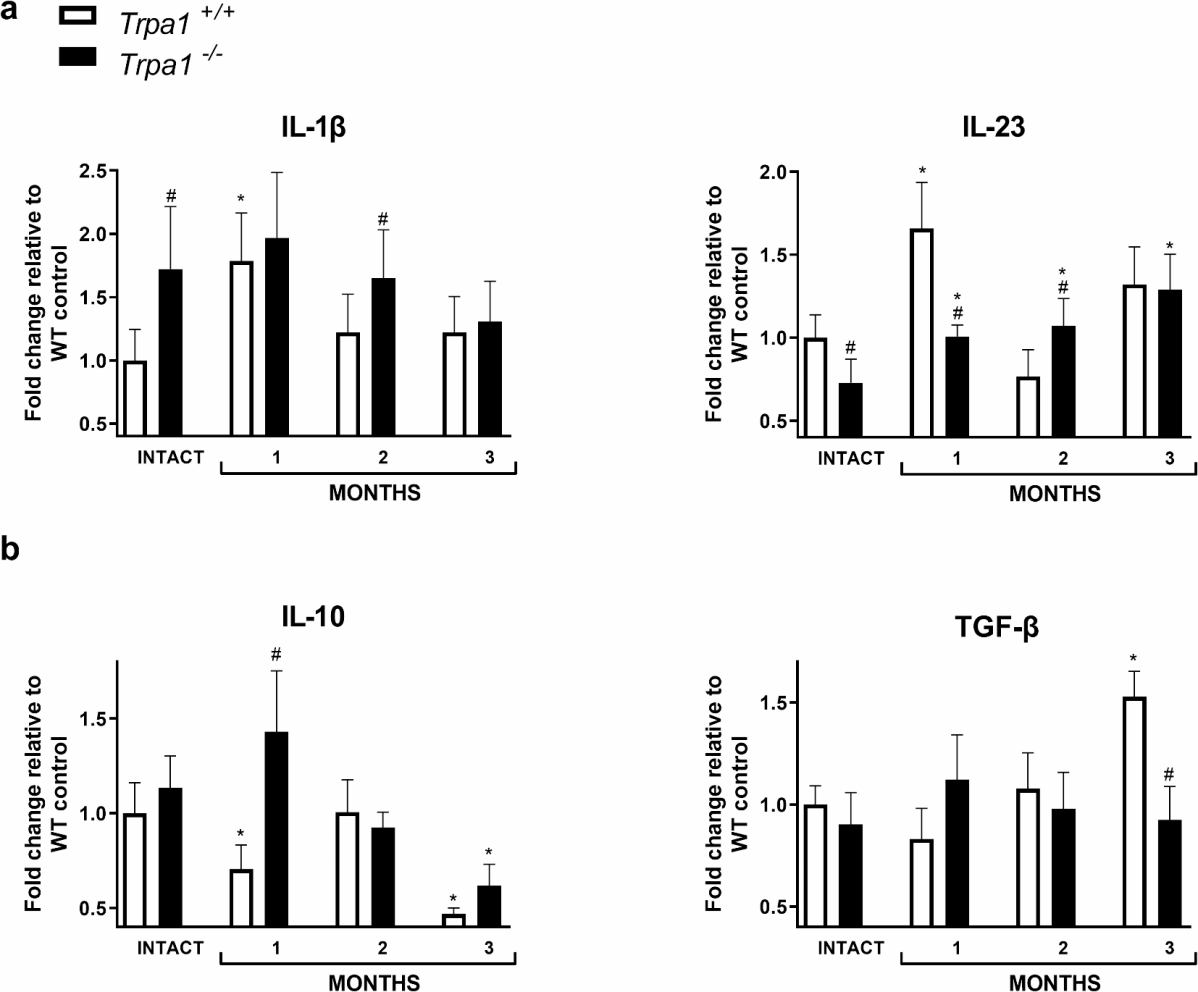
Smoking-induced mRNA expression changes of M1 and M2 cytokines in whole mouse lung homogenates. Each column represents mean ± SEM, n = 5/group, *g > 0.8 vs. respective control, #g > 0.8 vs. respective treatment, effect size analysis.

To explore the expression of M1 and M2-type cytokine mRNA levels of the mouse lungs gene expressional analysis has been performed. The M1-type cytokine IL-1β mRNA expression in the intact lungs of *Trpa1*^−/−^ mice was higher with large effect size compared to their *Trpa1*^+/+^ counterparts. After 1 month of CSE IL-1β was upregulated in *Trpa1*^+/+^ animals, but after 2-and 3 months of CSE this elevation decreased close to the initial expression level. The higher IL-1 level remained relatively stable throughout the 3-month CSE period with a large effect size at 2 months. IL-23 expression was lower in the intact *Trpa1*^−/−^ mouse lung. In *Trpa1*^+/+^ mice it increased after 1 month, then decreased and normalized to the baseline level after 2 and 3 months of CSE, respectively. In the lung of *Trpa1*^−/−^ mice CSE induced a gradual increase during the 3-month CSE paradigm being lower at 1 month and higher at 2 months compared to the *Trpa1*^+/+^ group (Fig. 4a).

The M2-type cytokines IL-10 and TGF-β expressions were the similar in the intact lungs of both genotypes. IL-10 decreased after 1 month and 3 months of CSE in *Trpa1*^+/+^ mice, which transiently normalized at 2 months. In the *Trpa1*^−/−^ group IL-10 decreased by the end of month 3 with large effect size compared to the intact value, but the expression was higher in comparison with the *Trpa1*^+/+^ mice only in month 1. TGF-β increased with large effect size by the end of month 3 only in *Trpa1*^+/+^, but not in *Trpa1*^−/−^ mice with also a genotype difference at this timepoint (Fig. 4b). TGF-β increase detected parallelly with *Trpa1* upregulation at 3 months of CSE. (Fig. 3b).

### CS extract induces TRPA1 upregulation in human lung 3D spheroids

**Fig. 5:**
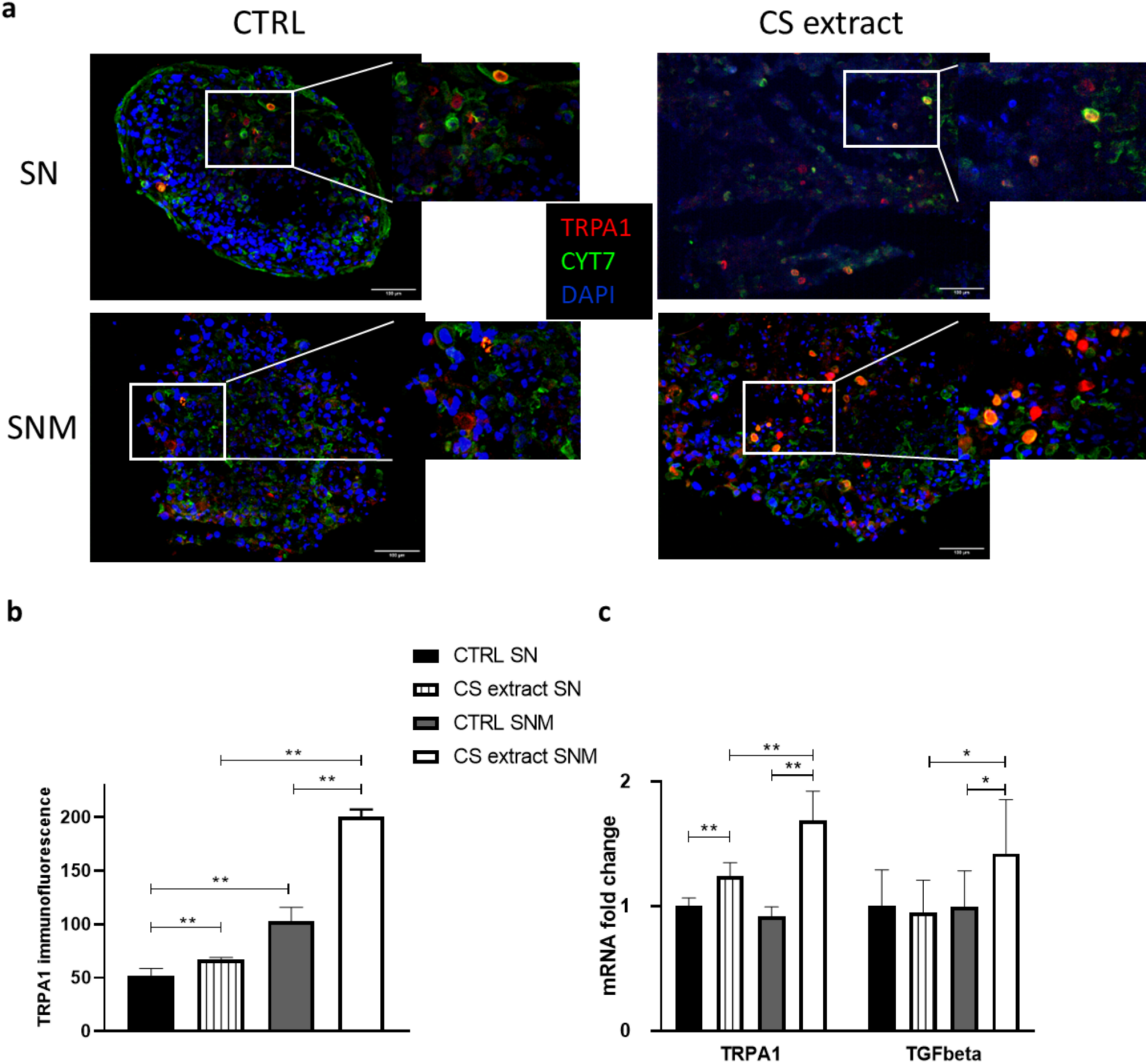
TRPA1 and TGF-β expressions in human lung spheroids. (**a**) Demonstrative pictures of TRPA1 immunopositivity of untreated and 24-h CS extract-treated SN (SAEC epithelial cells + NHLF fibroblasts) and SNM (+ primary macrophages) 3D human lung cultures. Red: TRPA1, green: cytokeratin7, blue: DAPI for nuclei. Scale bars, 100 µm, magnification 200x. (**b**) Quantification of TRPA1 protein with densitometry; each column represents mean ± SEM; n = 4–6 in the intact, 11–15 in the treated groups, **g > 0.8, effect size analysis. (**c**) TRPA1 and TGF-β mRNA expressions; n = 5, *g > 0.5, **g > 0.8, effect size analysis. Each column represents mean ± SEM.

To investigate the TRPA1 expression in the lung, a primary small airway epithelial cell (SAEC), normal human lung fibroblast (NHLF) and macrophage containing SNM spheroid and SAEC and NHLF containing aggregate SN were formed. TRPA1 immunopositivity (red) partially colocalized with cytokeratin7 (green) specific for epithelial cells was detected in spheroids. The TRPA1 signal density was greater in the SNM cultures compared to SN with a large effect size. 24-h CSE extract treatment increased TRPA1 expression in both the SN and SNM spheroids with much greater extent in the SNM cultures (Fig. 5a,b).

The immunopositivity results were basically supported by the mRNA expressional data demonstrating CS extract evoked upregulation in both cultures with greater extent in SNM spheroids with large effect size. TGF-β expression only increased in SNM cultures in response to CS extract treatment suggesting its macrophage origin (Fig. 5c).

## Discussion

These are the first data to show TRPA1 mRNA in mouse and human lung macrophages and demonstrate its functional expression. Furthermore, we described TRPA1 upregulation at mRNA and protein levels both in the mouse lung and human 3D lung tissues in response to chronic CS exposure, parallelly with M1 and M2 cytokine alterations.

Functional expression of TRPA1 in immune cells is inconclusive or controversial in the literature^49^ due to the technical problems with reliable detection methods such as antibody specificity^50^, isolation and culturing difficulties^51^. Minimal TRPA1 expression was found in murine Langerhans cells and skin macrophages^23^ and activated human macrophages infiltrating atherosclerotic and endometriosis lesions^16,52^. Others reported that TRPA1 mRNA was absent both in naïve and activated murine peritoneal macrophages, but the TRPA1 selective antagonists AP18 and HC-030031 inhibited nitrite production induced by LPS administration in macrophages^21,53^. Both qPCR and RNAscope ISH are useful techniques, but RNAscope is suitable to detect not only the expression but the tissue distribution of the mRNA, which can be co-localized with immunohistochemistry revealing cell type specific expression. In our experiments, *Trpa1* RNAscope ISH was used to visualize the mRNA in mouse lung tissue co-localized with *Iba1* RNAscope and CD68 immunohistochemistry.

In our experimental setup, with RNA-scope ISH we clearly demonstrated TRPA1 mRNA expression colocalizing with Iba1 in mouse lung macrophages. Although TRPA1 could not be detected in primary human lung macrophages by qPCR^40^ by others, our sensitive method demonstrated its presence also in human samples.

The airways are richly innervated by sensory afferents expressing TRPA1, having pro-tussive role^54^. Therefore, TRPA1 antagonists have become promising for cough treatment^55^. HC-030031 significantly inhibited acrolein-induced cough in guinea pigs^56^. GRC 17536 inhibited citrate-induced airway hyperreactivity in guinea pigs but did not prove to be effective in a Phase 2a clinical study^57,58^. GDC-0334 also inhibited cough response, airway smooth muscle hyperreactivity and oedema formation in rabbits, mice, guinea pigs and rats^59^, but was toxic^60,61^. TRPA1 is still in the focus of pharmacological research in inflammatory respiratory disorders, and several targeted studies are ongoing to evaluate its drug developmental potential^62,63^.

In vivo CSE under controlled repeatable conditions in rodents has been extensively used to study chronic inflammatory airway diseases. This model is important for translational research, especially with genetically engineered animals. In the present study, histopathological evaluation demonstrated duration-dependent chronic inflammation with edema formation, peribronchial, perivascular and parenchymal immune cell infiltration and emphysema in response to CSE in the lung of *Trpa1*^+/+^ mice, in agreement with our previous data. We demonstrated earlier that the same CSE protocol significantly altered airway function, decreased tidal volume, minute ventilation, peak inspiratory/expiratory flows in *Trpa1*^+/+^ but not in *Trpa1*^−/−^ mice. CSE induced emphysema after 2 months in wildtype mice, but only after 4 months in *Trpa1*^*−/−*^ ones^64^.

Macrophages and epithelial cells are among the first cell types of the respiratory system to encounter CS. Several studies have demonstrated the modulatory effects of CS on macrophage phenotypes, inflammatory cytokine expressions^65,66^ and potential TRPA1 involvement^67^. In our experiments, long-term CSE induced an initial increase of M1 (killing)- type IL-1β and IL-23 cytokines in the mouse lung homogenates, which shifted towards M2 (repair)-type IL-10 and TGF-β1 production by the end of the 3-month study in accordance with the literature data^68^. In our experimental setup *Trpa1* upregulation was detected after 2-and 3-months of CSE and a shift towards M2-specific phenotype dominated by TGF-β expression was observed at 3 months. Short-term CSE increased pro-inflammatory cytokine expression in rat and human macrophage cultures^69^. In acute exacerbation of COPD patients’ serum IL-23 increased compared to control group^70^. Downregulation of M1-, but elevation of M2-like macrophage phenotype was detected in the bronchoalveolar lavage fluid of smoking patients with COPD^34,71^.

Intact *Trpa1*^−*/*−^ mice had much higher baseline IL-1β, but lower IL-23 expression in the intact lungs. IL-1 β remained unaltered, while IL-23 gradually increased by CSE throughout the experiment, but M1-type cytokines were higher in *Trpa1*^−*/*−^ mice compared to *Trpa1*^+/+^ at the 2-month time point. The lower IL-23 expression not only in the intact lung but also 1 month after CSE in the *Trpa*^−*/*−^ group is supported by literature data showing reduced IL-23 production of dermal dendritic cells in *Trpa*^−*/*−^ mice in imiquimod-induced skin inflammation^72^. It was concluded that TRPA1 activation promotes dermal dendritic cells to produce IL-23, which in turn activates IL-17-producing T-cells.

The baseline levels of the M2 cytokines IL-10 and TGFβ were not influenced by the presence of TRPA1. IL-10 decreased after 1 month of CSE and even more by the end of the study in *Trpa1*^+/+^ mice, while in *Trpa1*^−/−^ ones it was much greater at the 1-month time point. TGFβ increased in the wild type group only by 3 months, but not in the knockouts. Literature data showed IL-10 expression in human, but not in mouse macrophages^73^ even after LPS stimulation^74^. It was upregulated in the BALF of mice after 12 days of CSE suggesting its predominant production by neutrophils^75^. IL-10 plays an important role in T helper cell polarization towards Th2 phenotype^76^. In agreement with our data, others also found that CSE decreased IL-10 levels in the BALF of CS-exposed mice suggesting Th1 dominance in the inflamed lung^77^. TGF-β regulates cell growth, morphogenesis, cell differentiation, apoptosis, tissue remodeling and fibrogenesis^78,79^. CS induced TGF-β release in the rat airways in response reactive oxygen species^80^ which are known TRPA1 activators. Plasma TGF-β levels were significantly higher in smoking COPD patients correlating with the number of cigarettes/years^81^. Our results showing TRPA1-dependent TGF-β production by the end of the 3-month experiment are supported by these data. TRPA1-mediated TGF-β downregulation was also shown in mouse primary eye fibroblasts^82^.

3D human lung spheroids can mimic tissue-like cell–cell interactions and the surrounding microenvironment. Our 3D spheroids, composed of SAEC, NHLF primary cell lines, and primary human macrophages, offer a more physiologically relevant model. They express a functional extracellular matrix that preserves tissue-specific architecture, protein expression, receptor profiles, and signaling pathways. Therefore, our results are close to human in vivo condition providing reliable results on TRPA1 expression of different cell types in the human pulmonary tissue.

In human 3D lung aggregates CS extract significantly increased the amount of TRPA1 mRNA and protein, which propose that smoking might induce non-neuronal TRPA1 expression. The receptor mRNA showed higher expression levels in SNM cultures compared to SM, suggesting that macrophages also contribute to this channel transcription in the lung. Unlike TRPA1, TGF-β mRNA elevated only in SNM aggregates suggesting that CS-driven lung damage is likely to be macrophage-dependent.

The mouse cytokine results were obtained from whole lung homogenates representing the overall inflammatory milieu not specific to certain cell types. However, the chronic CSE model proved to be translationally relevant for our major outcome measures such as TRPA1 and TGF-β expression as also shown by human lung 3D cell aggregates. The major limitation of this study is that we only detected TRPA1 at the mRNA level in the mouse lung since the antibody which showed specific signal in the human spheroids did not work on animal tissue. This problem with the TRPA1 antibodies has long been recognized as demonstrated in a comprehensive paper^50^, and supported by our previous experience^83,84^.

In conclusion, the functional TRPA1 expression in both mouse lung and human 3D spheroid with special emphasis on macrophages, as well as its smoking-induced upregulation parallelly with M1 and M2 cytokine alterations suggest its modulatory role in chronic airway inflammation and structural damage. Based on these promising results, further molecular research is needed on human lung tissues and 3D organoid cultures to determine TRPA1 as a potential pharmacological target in the airways.

## Methods

### Ethics

All experiments were carried out according to the European legislation (Directive 2010/63/EU) and Hungarian Government regulation (40/2013., II. 14.) on the protection of animals used for scientific purposes. All procedures were approved by the Ethics Committee on Animal Research of University of Pécs according to the Ethical Codex of Animal Experiments; license was given (BA02/2000-5/2011) and study reporting was in accordance to ARRIVE guidelines. Animals were kept in the animal housing facility of the Department of Pharmacology and Pharmacotherapy, Medical School, University of Pécs. The human blood collection used for macrophage culturing was approved by the Regional Research Ethics Committee of the University of Pécs Clinical Centre (license number: ETT 6444/2016) and all participants signed written consent in accordance with the Declaration of Helsinki. Regarding human lung tissue samples, archived paraffin embedded tissue blocks, originally harvested for pathological examinations were obtained from the Department of Pathology, Medical School, University of Pécs.

### Animals

Experiments were performed on male *Trpa1*^−/−^ mice and their *Trpa1*^+/+^ counterparts (8 weeks, 20–25 g; n = 4–5/group) housed and bred in the animal facility of the Department of Pharmacology and Pharmacotherapy, Medical School, University of Pécs under standard conditions (temperature 24–25 °C and provided with rodent chow and water ad libitum*. Trpa*^+*/−*^-deleted mice generated on the C57Bl/6 background were kindly provided by Prof. P. Geppetti (University of Florence, Italy). These mice were generated and characterized as described previously^85^. Offspring were genotyped and homozygous mice were selected for further breeding. Stable homozygous lines of *Trpa1*^*−/−*^ and *Trpa1*^+*/*+^ were successfully generated after 4 generations. *Trpa1*^+/+^ C57BL/6 mice are carrying two alleles of the wild type *Trpa1*, while *Trpa1* deficient (*Trpa1*^−/−^) mice were generated through targeted deletion of a genomic region encoding the pore-loop domain of the ion channel. During in vivo experiments, the depth of anesthesia is routinely checked by the absence of a reflex response to a mechanical stimulus applied with forceps on the hind legs.

### Mouse lung cell isolation and sorting

*Trpa1*^+/+^ male mice were deeply anesthetized with i.p. 1% sodium pentobarbital (70 μL/10 g). After abdominal aorta intersection, mice were perfused through the right ventricle with 10 ml of phosphate-buffered saline (PBS) to reduce lung blood content of the lung. For lung cell isolation 3 ml trypsin was poured into the trachea to initiate the fine digestion, then 10 ml PBS to eliminate the trypsin. Finally, the lung was filled with 3 ml of collagenase-dispase solution (3 mg/ml collagenase (Sigma-Aldrich, St. Louis, MO, USA) 1 mg/ml dispase (Roche F. Hoffmann-La Roche Ltd. Basel, Switzerland) and 1U/µl DNAse I (Sigma-Aldrich) through the trachea.

Lungs were then removed and cleaned from connective tissue. After the dissection of pulmonary lobes into smaller pieces, tissue was further digested for 50 min with 10 ml collagenase-dispase while continuous stirring was performed. The digested single cell solution was then filtered using a 70 μm cell-strainer (BD Becton, Dickinson and Company Franklin Lakes, NJ, USA) and was labeled with anti-CD45-FITC antibody^86^. Cell sorting was performed on BD FACSAria III (BD Life Sciences, San Jose, USA) cell sorter and the CD45 + cell population was collected. The purity of sorted cell population was above 99%.

### Primary cultures of mouse peritoneal macrophages

Peritoneal macrophages were isolated from the peritoneal cavity of male *Trpa1*^+/+^ mice (8 weeks, 20–25 g, n = 5) 4 h after i.p. lipopolysaccharide (LPS) endotoxin injection (300 µl of 300 µg/ml solution per animal, Sigma-Aldrich, St. Louis, MO, USA). Mice were terminated under ketamine- xylazine anesthesia (5 mg/kg) and the abdominal cavity was leached with 3 ml cell culture medium (RPMI 1640, Sigma, St. Louis, MO, USA) supplemented with 10% fetal bovine serum (Sigma, St. Louis, MO, USA) under sterile conditions. After cell counting with hemocytometer the lavage fluid was transferred into a 24-well plate and kept at 37 °C with 5% CO_2_ for 12 h.

### In vivo cigarette smoke exposure and tissue harvesting

We have earlier characterized the smoke exposure duration-dependent pathophysiological alterations in the mouse lung up to 6 months. Based on these earlier results, the most extensive inflammatory reaction with substantial peribronchial/ perivascular and interstitial inflammatory cell infiltration, damaged and irregular bronchial and epithelial cell layer developed at the 3-month-timepoint and was converted to tissue damage later^87^. Therefore, 3 months of CSE was selected in the present experimental setup.

*Trpa1*^*−/−*^ mice and their *Trpa1*^+*/*+^ counterparts were exposed to full-body cigarette smoke exposure (CSE) with the help of two-port TE-2 whole-body smoke exposure chamber (Teague Enterprise, USA). CSE was performed according to the protocol described previously (Kemény et al., 2017) using unfiltered 3R4F research cigarettes (Kentucky Tobacco Research and Development Center, University of Kentucky). Each mouse was exposed to 2 cigarettes for 10 min with a puff duration of 2 s and a puff frequency of 1/min/cigarette. Mice were exposed to smoke for 30 min followed by a 30 min of ventilation period, two times a day, 10 times a week for 1, 2 and 3 months. The total particulate matter (TPM) in the smoking chamber was 154.97 ± 5.18 mg/m^3^, with 9.86 ± 0.33 mg/m^3^ nicotine and 147.57 ± 4.93 ppm carbon-monoxide. TPM was determined every week. Control mice were exposed to room air for the same duration.

Animals were terminated by cervical dislocation under ketamine-xylazine induced anesthesia (5 mg/kg) after 1, 2 or 3 months of CSE. The lungs were removed, and one part was stored in TRI-reagent (Thermo Fischer Scientific, Waltham, MA, USA) for molecular biology experiments, the other part was placed to 6 V/V% formaldehyde solution (Szkarabeusz Ltd.; Pécs, Hungary) for immunohistochemical analysis.

### Ratiometric technique of intracellular free calcium concentration ([Ca^2+^]_i_) measurement

Peritoneal macrophages were stained for 30 min at 37 °C with 1 μM of fluorescent Ca^2+^ indicator dye, fura-2-AM (Molecular Probes) in a buffer solution containing (in mM): NaCl, 122; KCl, 3.3; CaCl_2_, 1.3; MgSO_4_, 0.4; KH_2_PO_4_ 1.2; HEPES, 25; glucose, 10; (pH 7.3). A 5-min washing period followed the dye loading in extracellular solution (ECS, containing in mM: NaCl, 160; KCl, 2.5; CaCl_2_, 1; MgCl_2_, 2; HEPES, 10; glucose, 10; pH 7.3). ECS was gravity fed to the cells using a fast step perfusion system (VC-77SP, Warner Instrument Corporation, Harvard Apparatus GmbH, Germany). Calcium transients of macrophages to ECS, vehicle control and AITC were examined with microfluorimetry as described earlier^88^. Fluorescence images were taken with an Olympus LUMPLAN FI/ × 20 0.5 W water immersion objective and a digital camera (CCD, SensiCam PCO, Germany), connected to a computer. Cells were illuminated alternately with 340 and 380 nm light generated by a monochromator (Polychrome II., Till Photonics, Germany) with the control of Axon Imaging Workbench 2.1 (AIW, Axon Instruments, CA). Emitted light was measured at 510 nm. The R = F340/F380 was monitored (rate 1 Hz) continuously. Baseline was adjusted to R = 0 and the peak magnitude of ratiometric responses was calculated.

### Preparation of cigarette smoke extract

Cigarette smoke extract solution was prepared as previously described^89^. The smoke of two Kentucky Research cigarettes (type 3R4F, College of Agriculture, University of Kentucky; Lexington, KY) was bubbled through 10 ml of FBS-free RPMI medium (Lonza, Basel, Switzerland) using a peristaltic pump (Harvard Apparatus, P-1500, Holliston, USA) at a flow rate of 550 ml/min for 3 min. The CS extract solution was filtered through a 0.22 μm diameter filter (TPP, Trasadingen, Switzerland) and considered as 100% solution, which was diluted with cell culture media to 0.5% concentration and applied to cells for further experiments. The pH values of the CS extract solutions were measured each time with pH 7.28 average value. The freshly prepared CS extract solutions were used within 30 min and the CS extract treatment was for 24 h in all cases.

### RNAscope in situ hybridization

RNAscope in situ hybridization (ISH) was performed on 3 µm thick paraformaldehyde fixed paraffin embedded (FFPE) healthy lung tissue and on the lungs of male *Trpa1*^+*/*+^ mice (4–5 weeks, n = 2). Animals were euthanized under deep anesthesia with urethane overdose (2.4 g/kg) of perfused transcardially with ice-cold 0.1 M phosphate-buffered saline (pH = 7.4) followed by 4% paraformaldehyde (Sigma-Aldrich, Saint Louis, USA) in Milloning buffer (pH = 7.4). Dissected lungs were postfixed for 72 h at 4 °C, embedded in paraffin using standard procedures and sections were cut using a sliding microtome (HM 430, Thermo Fisher Scientific, USA). The FFPE human lung tissue sample was obtained from post-surgical diagnostics of a lung adenocarcinoma patient after a pathological examination and specification of a healthy tissue section. After sectioning, slides were stored at 4 °C until further use.

After deparaffinization and rehydration, tissues were processed using the RNAscope Multiplex Fluorescent Reagent Kit v2 (Advanced Cell Diagnostics, Newark, CA, USA), according to the manufacturer’s instructions. Since the CD68 staining, which is a good marker for both mouse^90^ and human^91^ macrophages in standard immunohistochemistry did not work on the mouse samples processed for RNAscope, we used the Iba1 marker for alveolar macrophages^92^. Sections were hybridized with probes specific to mouse *Trpa1* (ACD, Cat.No. 400211-C2) and *Iba1* (ACD, Cat. No 319141) or human *TRPA1* (ACD, Cat.No 400211). Fluorescein for *Iba1* mRNA and cyanine 3 for *Trpa1* and *TRPA1* mRNA (1:750) were selected as fluorophores. RNAscope 3-plex mouse (ACD, Cat. No. 320881) or human (ACD, Cat. No. 320861) positive control probes specific to RNA polymerase II subunit A mRNA, peptidyl-prolyl cis–trans isomerase B mRNA (cyanine 3) and ubiquitin mRNA (cyanine 5) and 3-plex negative control probe (ACD, Cat. No. 320871) designed to bacterial 4-hydroxy-tetrahydrodipicolinate reductase were used in the experiments. After RNAscope ISH, human sample slides were subjected to an immunofluorescent labeling using monoclonal mouse anti-human CD68 macrophage marker (Dako, Clone KP1, code no. M814). Sections were washed in PBS and incubated with the CD68 antibody overnight, diluted 1:1000 in 2% normal goat serum containing PBS at room temperature (RT). After 3 × 15 min washing in PBS, sections were incubated for 3 h at RT with Alexa Fluor 488-conjugated goat anti mouse IgG (Invitrogen Antibodies, Cat. No. A28175, 1:500) secondary antibody. Sections were counterstained with DAPI and mounted with ProLong Glass Antifade Mounting Medium (Thermo Fisher Scientific, Waltham, MA, USA) for confocal imaging. Fluorescent images were taken with a Nikon Eclipse Ti2-E confocal microscope with 40 × and 60 × objectives.

Virtual colors were selected to depict fluorescent signals: blue for DAPI, green for CD68 (A488) and *Iba1* (FITC), red for *Trpa1* and *TRPA1* (Cyanine 3). Brightness/contrast adjustment with maximum intensity of separate channels were processed using FIJI (version 1.53c, NIH, USA).

### Isolation and induction of human peripheral macrophages

Monocytes were isolated from blood samples of healthy volunteers obtained from the National Blood Bank, Regional Centre Pécs. Briefly, human blood (20 ml/person) was collected from the median cubital vein of volunteers in blood collection tubes (BD Vacutainer, SST: BD SST™ Tubes with Silica Clot Activator and Polymer Gel, Franklin Lakes, NJ, USA). The peripheral blood mononuclear cells (PBMCs) were isolated by Ficoll-Paque PREMIUM (GE Healthcare, Sigma-Aldrich, St. Louis, USA) according to the manufacturer’s protocol. Monocytes were obtained by positive selection using magnetic-activated cell sorting (MACS, Miltenyi Biotec, GmbH, Bergisch Gladbach, Germany) and microbeads conjugated with anti-human CD14 antibody. CD14-positive cells were sorted with a magnetic column MACS MultiStand, OctoMACS (Miltenyi Biotech GmbH, Bergisch Gladbach, Germany). The purity of the isolated monocyte population was > 85%. Differentiation of macrophages was induced using 10 nM Phorbol 12-myristate 13-acetate (Sigma-Aldrich, Saint Louis, USA) at 37 °C with 5% CO_2_ for 5 h.

### Primary human lung cell cultures and 3D human lung tissue model

Mucin producing primary small airway epithelial cells (SAEC, Cat No: CC-2547) and normal human lung fibroblasts (NHLF, Cat No: CC-2512) with long branching processes and gap junctions were purchased from Lonza (ATCC, American Type Culture Collection, Rockville, MD), while the primary macrophages have been isolated and differentiated from the blood samples of 6 healthy volunteers by our research group. Samples have not been pooled from the different donors. NHLF is negative for von Willebrand Factor Expression/Factor VIII, cytokeratin 18 and 19, and alpha smooth muscle actin, while SAEC stains positive for cytokeratin 19. Cells were cultured using the manufacturer’s recommended media (Small Airway Growth Medium (SAGM, Fibroblast Growth Medium/FGM) and maintained under standard cell culture conditions (37 °C, 5% CO_2_).

3D lung tissue aggregates, also known as spheroids, were prepared on the bases of a previously granted patent (P0900819)^93^. For the experiments, two types of 3D lung cell aggregates were prepared: the SAEC and NHLF containing SN, and the SAEC, NHLF and primary human macrophage-containing SNM cultures. Cells were mixed for SN 3D aggregates in 50% NHLF, 50% SAEC proportion, and for SNM with the ratio of 2:2:1 of NHLF, SAEC and macrophages respectively. SN were cultured in SAGM: FGM cell culture media mixed in a 1:1 ratio and the SNM were maintained in a SAGM:FGM:RPMI media mixed in a 2:1:1 ratio. Cell suspensions were seeded on a 96-well U-bottom plate (Corning, New York, USA), centrifuged at 600 g for 10 min at room temperature, and incubated overnight under standard cell culturing conditions (37 °C, 5% CO_2_).

### RNA isolation and RT-qPCR

Intact, 1, 2 and 3 months CSE treated *Trpa1*^+*/*+^ and *Trpa1*^−*/*−^ male mice (n = 5/group) were anesthetized intraperitoneally with ketamine-xylazine (5 mg/kg), and after cervical dislocation, the trigeminal ganglia, nasal mucosa and the lungs of the mice were removed. Both tissues and 3D aggregates were then homogenized in 1 ml TRI Reagent. RNA was purified from the aqueous phase using the Direct-zol RNA MiniPrep (Cat. No. R2052; Zymo Research, Irvine, CA, USA) for tissue samples and MicroPrep (Cat. No. R2062; Zymo Research, Irvine, CA, USA) kits according to the manual. The quantity and quality of the extracted RNA were assessed on Nanodrop ND-1000 Spectrophotometer V3.5 (Nano-Drop Technologies, Inc., Wilmington, DE, USA). 200 ng of total RNA was reverse transcribed using Maxima First Strand cDNA Synthesis Kit (Cat. No. K1642, ThermoScientific, Santa Clara, CA, USA), and the qPCR amplification was performed using SensiFAST SYBR Green Lo-ROX Kit (Cat. No. BIO-94020B; Biocenter Ltd., Szeged, Hungary). Gene expression analyses were performed using Agilent, Stratagene Mx3000P equipment using β-actin as reference gene. Primers of similar efficiencies were considered when fold change values were calculated. Fold changes are expressed as 2^−ΔΔCq^ followed by log transformation and mean centering described by Willems et al.^94^. The sequences of the primers used in the gene expression analysis are listed in Table 1.

**Table 1:** Table of RT-qPCR primer sequences.

Species	Gene symbol	Primer sequence
Murine	Trpa1	Forward: 5′-ATCCAAATAGACCCAGGCACG-3′
		Reverse: 5′-CAAGCATGTGTCAATGTTTGGTACT-3′
	Il1β	Forward: 5′-TTCAGGCAGGCAGTATCACTC-3′
		Reverse: 5′-GAAGGTCCACGGGAAAGACAC-3′
	Il23	Forward: 5′-AGCGGGACATATGAATCTACTAAGAGA-3′
		Reverse: 5′-GTCCTAGTAGGGAGGTGTGAAGTTG-3′
	Tgf-β	Forward: 5′-GCCCTGGATACCAACTATTGC-3′
		Reverse: 5′-AGCTGCACTTGCAGGAGCG-3′
	Il10	Forward: 5′-GGTAGAAGTGATGCCCCAGGC-3′
		Reverse: 5′-CTATGCAGTTGATGAAGATGTC-3′
	β-actin	Forward: 5′-TTCCAGCCTTCCTTCTTGGG-3′
		Reverse: 5′-ACGGATGTCAACGTCACACT-3′
Human	TRPA1	Forward: 5′-GCAGAAATACCGGCTGAAGG-3′
		Reverse: 5′-GGC TAT CATCATCCTCTGTCTCA-3′
	β-actin	Forward: 5′-GCGCGGCTACAGCTTCA-3′
		Reverse: 5′-CTTAATGTCACGCACGATTTCC-3′
	TGF-β	Forward: 5′-GACATCAACGGGTTCACTACC-3′
		Reverse: 5′-CGTGGAGCTGAAGCAATAGTT-3′

### Immunohistochemistry and immunofluorescence staining

*Trpa1*^*+/+*^ and *Trpa1*^*−/−*^ mice were euthanized under deep ketamine- xylazine anesthesia (5 mg/kg). Lungs were removed and post-fixed in 4% paraformaldehyde. Serial 4 µm paraffin-embedded sections were prepared using a sliding microtome (HM 430, Thermo Fisher Scientific, USA). After routine deparaffination, rehydration and antigen retrieval, sections were treated with 1% H_2_O_2_ in Tris for 30 min. After appropriate washing, nonspecific antibody binding was blocked with 5% bovine serum albumin (BSA; Vector Laboratories, Burlingame, CA, USA). Sections were incubated with polyclonal rabbit anti-mouse CD68 (dilution: 1:2000, cat.no. ab125212, Abcam, Cambridge, UK) and peroxidase linked anti-rabbit IgG (1:200, Histols-R 30011.R500, Histopathology Ltd., Pécs, Hungary). CD68 labeled cells were stained with 3,3′-diaminobenzidine tetrachloride (Histols-DAB, Histopathology Ltd., Pécs, Hungary). Slides were visualized using Mantra quantitative pathology workstation (PerkinElmer, Waltham, USA) and analysed by inForm analysis software (Akoya Biosciences, Marlborough, Massachusetts, USA). Evaluation of DAB CD68-positive cell density of the *Trpa1*^*−/−*^ and *Trpa1*^+*/*+^ mouse lung tissue after CSE was performed using five tissue sections/group, and 20 images/section.

SN and SNM aggregates were carefully removed from the 96-well cell culture plate, were embedded into Cryomount embedding medium (Histolab, Finland) and were stored at -80 °C until further use. 8 μm sections were prepared using a Leica cryostat (CM1950, Leica, Wetzlar, Germany). After drying, sections were fixed in cold acetone (Molar Chemicals Kft., Hungary) for 10 min. The fixed sections were blocked in PBS containing 5% BSA (Sigma Aldrich, St. Louis, USA) for 20 min. For TRPA1 protein detection sections were labelled with rabbit polyclonal anti-TRPA1 (1:1000, ab62053, Abcam, Cambridge, UK). After washing steps, the secondary anti-rabbit polyclonal IgG Alexa Fluor 647 goat antibody (1:200, #A-21245, Termo Fisher Scientifc, Waltham MA) was added to the sections for 45 min. Mouse cytokeratin was visualized using monoclonal anti- cytokeratin7 antibody (1:100, M7018, Dako Denmark, Glostrup, Hovedstaden, Denmark) and polyclonal anti-mouse IgG Alexa Fluor 488 conjugated antibody secondary antibody (1:200, #A-11029, Termo Fisher Scientifc, Waltham MA) produced in donkey. Nuclei were visualized with DAPI (1:1000, Serva, Heidelberg, Germany). Images were captured with a confocal microscope (Zeiss LSM 710) and fluorescence images were edited and staining intensities were measured using FIJI (version 1.53c, NIH, USA) image editing software.

### Statistical analyses

The difference between the experimental groups has been determined by Hedges’ g corrected effect size analysis^95^. Results are displayed and discussed based on the effect sizes, however the results of conventional statistical analyses are shown in the Suppl. Table 1–5. In the case of ratiometric technique, paired Student’s t-test was used. Immunopositivity and gene expression data were analyzed by one-way ANOVA followed by Tukey’s multiple comparisons test, while the differences between cytokine levels were determined by two-way ANOVA followed by Tukey’s multiple comparisons test.

Data are presented as mean ± SEM in all cases together with medium (g > 0.5) and large (g > 0.8) effect sizes, except in the case of Fig. 4, where only the large (g > 0.8) effect sizes are displayed.

## Electronic supplementary material

Below is the link to the electronic supplementary material.

Supplementary Material 1

## Data Availability

The datasets used and/or analysed during the current study available from the corresponding author on reasonable request.
