# Supplementary material for: The TRPA1 cation channel is upregulated by cigarette smoke in mouse and human macrophages modulating lung inflammation
Source: Sci Rep. 2025 Mar 27;15:10661. doi: 10.1038/s41598-025-95662-y (PMC11950515; doi:10.1038/s41598-025-95662-y)
Supplement: Supplementary file 1 — Supplementary Material 1 [file 41598_2025_95662_MOESM1_ESM.pdf]

Supplementary table 1. Comparison of TRPA1 expression of Trpa1$^{+/+}$ mouse lungs after 1, 2 and 3 months of smoke exposure. The table shows the statistics performed using the means with SD of n=5-9 mice/group. p<0.05 (ordinary one-way ANOVA followed by Tukey's multiple comparisons test) was considered significant indicated by * and effect size was calculated using Hedges' *g*.

Group comparisons	Intact	1 month of CSE	2 months of CSE
1 month of CSE			
p value	0.9999		
Hedges' *g*	0.17		
2 months of CSE			
p value	0.0713	0.1772	
Hedges' *g*	1.1 ↑↑	0.92 ↑↑	
3 months of CSE			
p value	0.0753	0.1779	>0.9999
Hedges' *g*	1.74 ↑↑	1.44 ↑↑	0.02

Supplementary figure 1. Expression of Trpa1. A. Trpa1 mRNA levels in intact mouse tissue detected by qPCR. Each column represents mean±SEM, n=4.

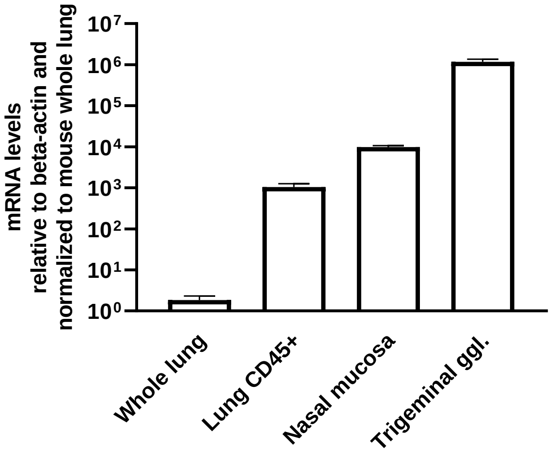

Supplementary table 2. Comparison of CD68 immunostaining demonstrating macrophages in intact and cigarette smoke exposed mouse lung tissues. The table shows the statistics performed using the means with SD of n=5 tissue sections/group, 20 images/section. $p<0.05$ (two-way ANOVA followed by Tukey's multiple comparisons test) was considered significant indicated by * and effect size was calculated using Hedges' *g*.

Groups	Comparisons of CD68 immunostaining of CSE treated Trpa1$^{+/+}$ mice		
	Intact	1 month of CSE	2 months of CSE
1 month of CSE<br>*p* value<br>Hedges' *g*	 0.0048**<br>1.26 ↑		
2 months of CSE<br>*p* value<br>Hedges' *g*	 0.2495<br>1.42 ↑	 0.7989<br>0.46	
3 months of CSE<br>*p* value<br>Hedges' *g*	 0.0028**<br>1.67 ↑	 >0.9999<br>0.02	 0.7133<br>0.6 ↑

Groups	Comparisons of CD68 immunostaining of CSE treated Trpa1$^{-/-}$ mice		
	Intact	1 month of CSE	2 months of CSE
1 month of CSE<br>*p* value<br>Hedges' *g*	 0.9998<br>0.14		
2 months of CSE<br>*p* value<br>Hedges' *g*	 0.9892<br>0.35	 0.9998<br>0.16	
3 months of CSE<br>*p* value<br>Hedges' *g*	 >0.9999<br>0.07	 0.9949<br>0.2	 0.9295<br>0.38

	Intact	1 month of CSE	2 months of CSE	3 months of CSE
Comparisons of CD68 immunostaining of CSE treated Trpa1$^{+/+}$ to Trpa1$^{-/-}$ mice<br>*p* value<br>Hedges' *g*	 0.06<br>1.37 ↑↑	 0.946<br>0.3	 >0.9999<br>0.04	 0.9995<br>0.14

Supplementary table 3. Comparison of IL-1β mRNA expression of intact and cigarette smoke exposed mouse lung tissues. The table shows the statistics performed using the means with SD of n=5. $p<0.05$ (two-way ANOVA followed by Tukey's multiple comparisons test) was considered significant indicated by * and effect size was calculated using Hedges' *g*.

Groups	Comparisons of IL-1β mRNA expression of CSE treated Trpa1$^{+/+}$ mice		
	Intact	1 month of CSE	2 months of CSE
1 month of CSE			
p value	0.8141		
Hedges' *g*	1.1 ↑↑		
2 months of CSE			
p value	0.9999	0.9604	
Hedges' *g*	0.36	0.74↓	
3 months of CSE			
p value	0.9999	>0.9999	>0.9999
Hedges' *g*	0.37	0.75↓	0.0

Groups	Comparisons of IL-1β mRNA expression of CSE treated Trpa1$^{-/-}$ mice		
	Intact	1 month of CSE	2 months of CSE
1 month of CSE			
p value	0.9997		
Hedges' *g*	0.22		
2 months of CSE			
p value	>0.9999	0.9987	
Hedges' *g*	0.07	0.31	
3 months of CSE			
p value	0.9931	0.9119	0.9978
Hedges' *g*	0.45	0.69↓	0.44

	Intact	1 month of CSE	2 months of CSE	3 months of CSE
Comparisons of IL-1β mRNA expression of CSE treated Trpa1$^{+/+}$ to Trpa1$^{-/-}$ mice				
p value	0.8693	>0.9999	0.9913	>0.9999
Hedges' *g*	0.83 ↑↑	0.18	0.56 ↑	0.75 ↑

Supplementary table 4. Comparison of IL-23 mRNA expression of intact and cigarette smoke exposed mouse lung tissues. The table shows the statistics performed using the means with SD of n=5. p<0.05 (two-way ANOVA followed by Tukey's multiple comparisons test) was considered significant indicated by * and effect size was calculated using Hedges' *g*.

Groups	Comparisons of IL-23 mRNA expression of CSE treated Trpa1$^{+/+}$ mice		
	Intact	1 month of CSE	2 months of CSE
1 month of CSE<br>*p* value<br>Hedges' *g*	 0.217<br>1.35 ↑↑		
2 months of CSE<br>*p* value<br>Hedges' *g*	 0.9843<br>0.7 ↓	 0.0316*<br>1.77 ↓↓	
3 months of CSE<br>*p* value<br>Hedges' *g*	 0.9138<br>0.77 ↑	 0.8932<br>0.6 ↓	 0.4153<br>1.27 ↑↑

Groups	Comparisons of IL-23 mRNA expression of CSE treated Trpa1$^{-/-}$ mice		
	Intact	1 month of CSE	2 months of CSE
1 month of CSE<br>*p* value<br>Hedges' *g*	 0.9583<br>1.12 ↑↑		
2 months of CSE<br>*p* value<br>Hedges' *g*	 0.8866<br>1.27 ↑↑	 >0.9999<br>0.22	
3 months of CSE<br>*p* value<br>Hedges' *g*	 0.4057<br>1.37 ↑↑	 0.9564<br>0.79m ↑	 0.9894<br>0.51 ↑

	Intact	1 month of CSE	2 months of CSE	3 months of CSE
Comparisons of IL-23 mRNA expression of CSE treated Trpa1$^{+/+}$ to Trpa1$^{-/-}$ mice<br>*p* value<br>Hedges' *g*	 0.964<br>0.87 m↑↑	 0.2289<br>1.45 ↑↑	 0.9348<br>0.83 ↑↑	 >0.9999<br>0.07

Supplementary table 5. Comparison of IL-10 mRNA expression of intact and cigarette smoke exposed mouse lung tissues. The table shows the statistics performed using the means with SD of n=5. $p<0.05$ (two-way ANOVA followed by Tukey's multiple comparisons test) was considered significant indicated by * and effect size was calculated using Hedges' *g*.

Groups	Comparisons of IL-10 mRNA expression of CSE treated Trpa1$^{+/+}$ mice		
	Intact	1 month of CSE	2 months of CSE
1 month of CSE			
p value	0.9107		
Hedges' *g*	0.91 ↓↓		
2 months of CSE			
p value	>0.9999	0.9025	
Hedges' *g*	0.02	0.89 ↑↑	
3 months of CSE			
p value	0.3554	0.9721	0.3427
Hedges' *g*	2.05 ↓↓	1.13 ↓↓	1.95 ↓↓

Groups	Comparisons of IL-10 mRNA expression of CSE treated Trpa1$^{-/-}$ mice		
	Intact	1 month of CSE	2 months of CSE
1 month of CSE			
p value	0.9125		
Hedges' *g*	0.51 ↑		
2 months of CSE			
p value	0.9851	0.4179	
Hedges' *g*	0.71 ↓	0.96 ↓↓	
3 months of CSE			
p value	0.3851	0.0318*	0.8923
Hedges' *g*	1.61 ↓↓	1.5 ↓↓	1.38 ↓↓

	Intact	1 month of CSE	2 months of CSE	3 months of CSE
Comparisons of IL-10 mRNA expression of CSE treated Trpa1$^{+/+}$ to Trpa1$^{-/-}$ mice				
p value	0.999	0.0754	>0.9999	0.9983
Hedges' *g*	0.3654	1.32 ↑↑	0.27	0.7864 ↑

Supplementary table 6. Comparison of TGF-β1 mRNA expression of intact and cigarette smoke exposed mouse lung tissues. The table shows the statistics performed using the means with SD of n=5. p<0.05 (two-way ANOVA followed by Tukey's multiple comparisons test) was considered significant indicated by * and effect size was calculated using Hedges' *g*.

Groups	Comparisons of TGF-β1 mRNA expression of CSE treated Trpa1$^{+/+}$ mice		
	Intact	1 month of CSE	2 months of CSE
1 month of CSE			
p value	0.9949		
Hedges' *g*	0.61 ↓		
2 months of CSE			
p value	>0.9999	0.9578	
Hedges' *g*	0.24	0.67 ↑	
3 months of CSE			
p value	0.3173	0.0761	0.5098
Hedges' *g*	2.16 ↑↑	2.27 ↑↑	1.32 ↑↑

Groups	Comparisons of TGF-β1 mRNA expression of CSE treated Trpa1$^{-/-}$ mice		
	Intact	1 month of CSE	2 months of CSE
1 month of CSE			
p value	0.9768		
Hedges' *g*	0.52 ↑		
2 months of CSE			
p value	>0.9999	0.9982	
Hedges' *g*	0.21	0.32	
3 months of CSE			
p value	>0.9999	0.9875	>0.9999
Hedges' *g*	0.06	0.45	0.14

	Intact	1 month of CSE	2 months of CSE	3 months of CSE
Comparisons of TGF-β1 mRNA expression of CSE treated Trpa1$^{+/+}$ to Trpa1$^{-/-}$ mice				
p value	0.9999	0.9018	0.9999	0.1785
Hedges' *g*	0.34	0.69 ↑	0.24	1.86 ↓↓

Supplementary table 7. Comparisons of TRPA1 and TGF-β1 expression changes in control and cigarette smoke-exposure (CSE) treated 3-dimensional SN/SNM spheroids. The table shows the statistics performed using the means with SD of n=4-15. p<0.05 (ordinary one-way ANOVA followed by Tukey's multiple comparisons test) was considered significant indicated by * and effect size was calculated using Hedges' *g*.

Groups	TRPA1 immunostaining		
	Non-smoking SN	Smoking SN	Non-smoking SNM
Smoking SN			
p value	0.5		
Hedges' *g*	1.29 ↑↑		
Non-smoking SNM			
p value	0.0039**		
Hedges' *g*	2.43 ↑↑		
Smoking SNM			
p value		<0.0001***	<0.0001***
Hedges' *g*		6.29 ↑↑	3.63 ↑↑

Groups	TRPA1 mRNA expression		
	Non-smoking SN	Smoking SN	Non-smoking SNM
Smoking SN			
p value	0.6065		
Hedges' *g*	1.22 ↑↑		
Non-smoking SNM			
p value	0.9747		
Hedges' *g*	0.52 ↑		
Smoking SNM			
p value		0.1354	0,0053**
Hedges' *g*		1.11 ↑↑	2.0 ↑↑

Groups	TGFβ mRNA expression		
	Non-smoking SN	Smoking SN	Non-smoking SNM
Smoking SN			
p value	0,9994		
Hedges' *g*	0.09		
Non-smoking SNM			
p value	>0.9999		
Hedges' *g*	0.00		
Smoking SNM			
p value		0.7323	0.7933
Hedges' *g*		0.60 ↑	0.52 ↑
